# Risk Factors of Breast Problems in Mothers and Its Effects on Newborns

**DOI:** 10.5812/ircmj.8582

**Published:** 2014-06-05

**Authors:** Hassan Boskabadi, Mahjoubeh Ramazanzadeh, Maryam Zakerihamidi, Farzaneh Rezagholizade Omran

**Affiliations:** 1 Department of Pediatrics, Mashhad University of Medical Sciences, Mashhad, IR Iran; 2 Department of Midwifery, Bardsir Branch, Islamic Azad University, Bardsir, IR Iran; 3 Department of Midwifery, College of Nursing and Midwifery, Mashhad University of Medical Sciences, Mashhad, IR Iran; 4 School of Medicine, Mashhad University of Medical Sciences, Mashhad, IR Iran

**Keywords:** Breast, Mastitis, Nipple, Risk Factors, Neonatal Complications

## Abstract

**Background:**

During lactation, especially the first few days after birth, some breast problems can cause pain and inadequate milk emptying.

**Objectives:**

This study aimed to investigate the risk factors of breast problems and their effect on neonatal complications.

**Patients and Methods:**

This case-control study was conducted on 566 infants referred to Mashhad Ghaem Hospital clinic (from 2008 to 2012) in Iran. Researchers filled out the questionnaire with the relevant neonatal and maternal information. They also recorded the mothers' breasts problems after examination. Then the infants were divided into two groups: Infants whose mothers complained from breast problems (case group), and the infants whose mothers did not have any breast problems (control group). Finally, two groups were compared with respect to the maternal and neonatal risk factors.

**Results:**

The two groups were matched on these variables: parity (P = 0.861), maternal weight (P = 0.577), education level (P = 0.807), pregnancy complications (P = 0.383), gestational age (P = 0.161), Apgar score (P = 0.530), birth weight (P = 0.090), infant gender (P = 0.439), and infant age (P = 0.152). Case group vs. control group showed significant differences regarding prenatal care, mode of delivery, lactation status and the letdown reflex, serum sodium, frequency of urination and defecation, new weight and supplementation (P < 0.05).

**Conclusions:**

According to our study, breast problems were fewer in mothers who had prenatal care, normal vaginal delivery, proper breastfeeding position, and let down reflex. Neonatal complications of breast problems include pathologic weight loss and decrease in the frequency of urination. Therefore, special attention to mother's breasts during pregnancy and in the early days of delivery, and their appropriate treatment may reduce breast problems and related neonatal complications.

## 1. Background

Breastfeeding is universally considered to be the best and the safest way of feeding neonates. Studies show that breast milk decreases the prevalence and severity of lower respiratory tract infections, acute otitis media, bacteriemia, bacterial meningitis, botulism, urinary tract infections and necrotizing enterocolitis in newborns. Also it is associated with increased cognitive development of infants (1-3). Furthermore, there are positive effects of breastfeeding after delivery for mothers, such as acceleration of uterine involution, contraception effect, and spacing between children. The psychological and mental benefits of breastfeeding are obvious. It provides not only a sense of tranquility and emotional dependence for the mother but also a biological and emotional basis for both the mother and her baby. Breastfeeding of a healthy infant occasionally turns into some challenges for the mother and her infant. Some of these challenges are predictable and some are not (4, 5). They can interfere with the process of lactation and exacerbate the maternal anxieties and worries (6, 7). Some of the prevalent problems are breast engorgement, plugged ducts, breast infection, breast abscess, and nipple fissures. These problems can cause pain, inadequate emptying of the breasts, and early weaning for mother (6-8). They may appear immediately after delivery or anytime during the lactation period (2). Several related studies have reported the incidence rate of 2-3% for mastitis, 25-85% for breast engorgement with plugged ducts, and 11-96% for breast fissure. Breast abscess co-occurs with mastitis in 5-11% of women (2-9). Inadequate breast emptying, improper breastfeeding techniques, using pacifiers and traditional nutritional supplements for the baby, as well as infant’s poor sucking are the risk factors that can account for the breasts’ problems (3, 7). Health personnel play a vital role in the prevention and management of these problems. They should be equipped with the sufficient knowledge and skills (6). Also, infants need adequate contact with the breast to have successful feeding, thus breast problems can undermine their effective contact (2). Therefore, examination of the mother’s breast, especially the areola and nipple, is important to identify the anatomic problems that may need treatment before or after delivery (10). In spite of studies on factors affecting lactation in underdeveloped and developed countries, the effect of breast problems on the infant's life has been somehow overlooked.

## 2. Objectives

In this study, we decided to investigate the risk factors of breast problems such as inverted nipple, nipple fissures and mastitis and their effect on causing neonatal complications.

## 3. Materials and Methods

A total of 566 infants admitted to Mashhad Ghaem Hospital clinic (a teaching hospital comprises 1000 beds) or its neonatal intensive care unit (NICU) from late April 2008 to late May 2012, were enrolled in this study. This state and general hospital has an active maternity ward and a neonatal level B3 ward with 25 beds. This study was conducted with the approval of the Ethics Committee of Mashhad University of Medical Sciences (MUMS) with the code number of 87913. Parental informed consent was obtained for each patient before enrollment in the study. After enrollment, one observer (a midwife) filled out a checklist consists of infant’s weight, laboratory tests, maternal and neonatal history. The results of the breast examination (performed by an expert midwife), and neonatal examination (performed by a neonatologist) were also recorded. Sampling was done using convenience sampling method. Twenty-four infants were excluded from the study because of the following reasons: prematurity (8 cases), formula-fed (6 cases), sepsis (1 case), multiple congenital anomalies (3 cases), having chromosomal or heart disease or being newborn (3 cases), having an Apgar score less than 7 at 1 minute (3 cases). Based on mothers’ breast examination, infants were divided into two groups: infants whose mothers had breast problems (case group) and infants whose mothers had not breast problems (control group). Sample volume is obtained 207 in each group by comparing two means formula of Dr. Vazirnejad study who has obtained the difference of with average weight of infants of mothers with healthy breast and those of ill breast mothers as 101 g and considering V = 1.65, v = 0.84. Since the number of participants in the experimental group can be 2 to 3 times the number of participants in the control group, 207 and 359 participants were studied in experimental and control groups, respectively (11). Criteria for breast problem were defined as the presence of one or more of the following problems: inverted nipple, nipple fissures or mastitis identified during the physical examination (4). Inverted nipple is referred to a nipple that is under the surface of the areola (12). Nipple fissure is a sore on mother's nipple, which can be in the form of cross, star and other shapes (10). Mastitis is the swollen and painful inflammation of the breast that may be associated with fever and weakness (13). Breast engorgement is defined as a large and heavy breast due to milk accumulation, increased fluid in breast tissues or increased blood flow in breast that usually occurs during the first week of lactation. In breast engorgement, the breasts are painful, hard, hot, swollen, red and even reddish so that it may interfere with the letdown reflex and in some cases the nipple may be stretched, so that it is difficult for the infant to keep enough breast tissue in the mouth (3). In these two groups, factors associated with the quality of lactation were investigated, including the number of feeding per day, duration of feeding in minutes, existence of letdown reflex, type of infant feeding (breast milk, formula feeding), and existence of traditional supplements (camel’s thorn, flixweed and sugar water). Neonatal parameters, including age, birth weight, new weight, Apgar scores, gestational age, gender, duration of feeding, frequency of urination and defecation per day and the first defecation time were recorded in neonatal information form.

 Maternal parameters, including age, weight, education, parity, pregnancy complications, mode of delivery, breast problems, techniques (position) of breastfeeding, let down reflex, the first breastfeeding time and the number of feeding per day were recorded for both groups.

Pregnancy complications consist of hypertension, diabetes mellitus, anemia, vaginal bleeding, preeclampsia, infections, malignancy, endocrine disorders, collagen vascular disorder, and epilepsy. The letdown reflex is the milk ejection reflex in response to baby suckling. The classic breastfeeding position or the cradle position was considered as the normal position. In the classic position, the mother’s same sided arm supports the infant at the breast on which it feeds, and the infant’s head is near the mother’s elbow. The Mother’s arm supports the baby along the back, and the infant is facing the mother, chest to chest. The lower lip is curved outwards, and the infant’s chin is attached to the mother’s chest (3). Regarding the data analysis, we proceeded according to the type of our study (case-control study). Statistical analysis was carried out using SPSS version 16.5. The group comparisons were assessed by the Pearson correlation test, Student’s t-test and analysis of variance (ANOVA) test. Test of ANOVA was used for comparing pregnancy complications (in case of normally distributed data) and nonparametric tests such as Spearman correlation test, Mann-Whitney test, and Kruskal–Wallis test were also used (in case of non-normally distributed data). Chi-square test was used to analyze the relationship between variables with nominal scales. In this study, P value < 0.05 was considered statistically significant.

## 4. Results

In this study, 207 infants of mothers with breast problems (cases) were compared with 359 infants of mothers without breast problems (control group). One hundred forty-six mothers in the case group had breast fissures, 89 mothers had inverted nipple and 46 mothers had mastitis. The rest of the case group (72 mothers) had two or three problems together.

 Statistical analysis showed that 59.8% of total infants were male and 40.2% were female. Also breast problems were seen in 32% of mothers with boys and 35% of mothers with girls. The mode of delivery was caesarean section in 60% and normal delivery in 40% of cases. In both groups (with and without breast problems), there were not statistically significant differences in these variables: parity (P = 0.861), maternal weight (P = 0.577), education level (P = 0.807), pregnancy complications (P = 0.383), gestational age (P = 0.161), Apgar score (P = 0.530), birth weight (P = 0.090), infant gender (P = 0.439), and infant age (P = 0.152). The two groups were matched on these variables (Table 1).

 In 310 infants (56%), a history of consumption of traditional supplements such as sugar water (59 infants), camel’s thorn (110 infants) and flixweed (14 infants) was observed. A total of 65% of infants of mothers with breast problems and 50% of infants of mothers without breast problems have mentioned a history of consumption of such supplements (P = 0.002) (Table 2).

A Significant difference was found between the mode of delivery (normal vaginal delivery vs. caesarean section) in two groups (with and without breast problems) whereas, breast problems were more in mothers who had caesarean section (Table 2, P = 0.007). Also, 35% of mothers with breast problems experienced problems in their labor compare to 18% in the control group (P = 0.003). Neonatal weight loss was more in mothers with breast problems (P = 0.013). The number of urination and defecation was fewer in case group, and a significant difference was seen between two groups (P = 0.001, Table 1). There was a delay or lack of let down reflex in 29% of mothers in the case group and natural let down reflex was reported significantly lower in mothers with breast problems (Table 2, P = 0.001).

**Table 1.** Characteristics of Case and Control Mothers and Infants^a,b^

Variables	Mothers With Breast Problems (Case)	Mothers Without Breast Problems (Control)	P Value
**Age, y**	8.2 ± 4.2	8.0 ± 4.4	0.718^c^
**Birth Weight, kg**	3.229 ± 0.474	3.1530 ± 0.5289	0.090
**New Weight, kg**	2.9452 ± 0.5333	3.1530 ± 0.5900	0.013
**First Minute APGAR Score **	9.0 ± 0.3	9.0 ± 0.3	0.530
**Time of First Breast-Feeding, h**	1.8 ± 0.2	3.0 ± 0.31	0.000^c^
**Number of Feeding, d**	10.9 ± 3.3	11.6 ± 3.3	0.049^c^
**Number of urination, d**	4.4 ± 1.1	5.4 ± 1.8	0.001^c^
**Number of Defecation, d**	3.4 ± 1.9	4.1 ± 1.9	0.001^c^
**Maternal age, y**	28 ± 5.9	26.3 ± 5.9	0.003^c^
**Parity**	1.9 ± 1.0	1.8 ± 1.0	0.861

^a^ Values are expressed as mean ± SD.

^b^ Comparisons between control and case groups were made using the Student's t test.

^c^ Mann-Whitney U test.

**Table 2.** Comparison of Maternal and Neonatal Variables in Two Groups^a^

Variables	Mothers With Breast Problems (Case)	Mothers Without Breast Problems (Control)	Chi-Square Test Results P Value	Odd Ratio
**Gender**	-	-	0.439	1.08
Male	108	181	-	-
Female	65	128	-	-
**Lactation Status**	-	-	0.001	2.58
Favorite	119	280	-	-
Unfavorite	72	56	-	-
**Let down Reflex **	-	-	0.001	1.87
Present	101	237	-	-
Absent	84	75		-
**Mode of Delivery**	-	-	0.007	1.25
NVD	104	95	-	-
CS	72	76	-	-
**Prenatal Care**	-	-	0.029	1.35
Yes	184	339	-	-
No	14	76	-	-
**History of Traditional Supplementation **	-	-	0.002	1.44
Consumption	132	174	-	-
No Consumption	71	178	-	-

^a^ Abbreviations: NVD, Normal Vaginal Delivery; CS, Caesarean Section.

## 5. Discussion

In the present study, we have determined that prenatal care, normal delivery, proper breastfeeding position, and exclusive breastfeeding reduce the risk of breast problems. Breast problems increase the risk of neonatal weight loss and its complications. The two studied groups were matched regarding the variables of parity, maternal weight, education level, pregnancy complications, Apgar score, birth weight, infant gender and neonatal age. These findings showed that the observed differences in two groups were related to the breast problems whereas infant factors were homogeneous in both groups.

 The most common breast problem in this study was breast fissure. Inverted nipples and mastitis were in the next level, and 30% of mothers have two or three problems together. Several studies have reported 2-3% incidence rate for mastitis, 11- 96% for breast fissure and breast abscess occurs in 5-11% of women with mastitis (5-8).

The results showed that mothers with breast problems had poor prenatal care. Therefore, the detection of some problems like inverted nipple and its appropriate treatment before child birth can reduce the problems of first days of lactation. Also an appropriate education with regard to breastfeeding challenges in the first days, would prepare mothers to have a successful breastfeeding (3).

 In our study, breast problems in mothers who had caesarean section was significantly more than mothers who had normal delivery (P = 0.007). Several studies have shown that caesarean section causes delays in breastfeeding, poor feeding conditions due to pain, and increase in neonatal problems like prematurity and transient tachypnea of the newborn, which lead to increase in breast problems and reduction in the chance of successful breastfeeding (3, 4). This study showed that breast problems would result in neonatal weight loss and hypernatremia.

These findings were consistent with other related studies (14, 15). The study of Cooper et al. also showed that 3 out of 5 mothers of infants admitted to the hospital for hypernatremia and dehydration had inverted nipples (16). In fact, weight loss up to 10% of initial weight can be caused by an imbalance between milk intake and infant's need (14). In another study about the risk factors for weight loss and hypernatremic dehydration, the breast problems have been mentioned as a predisposing factor (4).

Neonatal weight gain is one of the measures of the adequacy of infant feeding. Breast problems such as mastitis, inverted nipple and nipple fissures cause inadequacy of infant feeding. If such problems occur during lactation and cause poor breastfeeding, they can aggravate breast problems. Breastfeeding is the best source of infant nutrition, and inadequate nutrition would result in significant weight loss and hypernatremic dehydration. This dehydration can result in fatal complications, as expressed in Caglar’s study (14). This finding is valuable as we notice the rapid infant growth in the early weeks and losing the chance of receiving colostrum in the first days of life (17). In another cohort study in Iran done by Vazirinejad et al. it was shown that anatomical variations of the breast, including inverted nipples had adverse effects on breastfeeding, which confirmed previous findings (11). The findings also showed that the delay in breastfeeding was shorter in mothers who had no breast problems. Delay in breastfeeding probably leads to more breast engorgement and results in disturbance in the physiological processes of lactation (3). Newton considered early initiation and frequent feeding as one of the causes of decrease in breast engorgement after delivery (18). Dewey et al. reported a relationship between the delayed onset of lactation and breast problems in the day’s one, three, and seven after delivery (19). These findings indicate the importance of early feeding after delivery and decrease in breast problems such as engorgement (18). Based on our findings, supplementation was more in infants whose mothers had breast problems (P = 0.002). Traditional supplements like camel's thorn, flixweed, and sugar water have been reported in 58% of neonates that reflex the belief of our society about their effects. There is a public belief that some food which cause diarrhea may be effective in reducing jaundice. This belief has led to the consumption of camel, thorn, flixweed and similar substances in our culture. As we know, jaundice is a self-limiting process and consuming these substances and spontaneous improvement of jaundice make this belief more sound (20). In several of in vitro (21, 22) and human studies, no positive effect of these substances has been proven in neonates . They were also ineffective in controlling jaundice and weight loss (23, 24). In another study, delays in time of visit and even exacerbated jaundice have been reported by consuming these supplements (20).

In some studies, high body mass index and overweight of the mother were considered as risk factors for breastfeeding problems (25). In this study, maternal weight was higher in the case group, but no significant difference was found between two groups in terms of this variable.

 Improper breastfeeding position is another factor in our study that was obviously more in case group than control group (P = 0.001). Successful breastfeeding requires proper placement of nipple-areola complex in baby's mouth. If the mother does not have a proper position, the risk of nipple sores and breast fissures increases. Therefore the possibility of having a successful breastfeeding will reduce (3, 4). The absence of milk ejection reflex is one of our findings in the case group that was the outcome of breast problems and a sign of delay in letdown reflex. This factor has been effective in weight loss in most infants of the case group. In a study, the absence of this reflex has increased the risk of weight loss in infants (4).

Close collaboration between midwife and neonatologist and proper follow-up and comprehensive study of infants were the strengths of this study. Not following up the ill mothers’ destiny was the weakness of the study. According to our study, prenatal care, normal delivery, proper breastfeeding position, existence of let down reflex immediately after delivery and no consumption of traditional supplements such as sugar water, camel’s thorn, and flixweed would reduce the risk of breast problems. Breast problems increase the risk of hypernatremia, weight loss, and decrease in the number of urination. Training of pregnant mothers by health personnel, midwives and gynecologists can reduce such problems. The health personnel should pay attention to mother's breast problems such as nipple fissures, mastitis, and so on in the early days of delivery.
